# Advances in Tissue-Agnostic Targeting in Cancer Therapeutics: Current Approvals, Challenges, and Future Directions

**DOI:** 10.32604/or.2025.067791

**Published:** 2025-10-22

**Authors:** Matthew Rubinstein, Madeline Lauren Hong, Rishi Kumar Nanda, Daniel Thomas Jones, Hazem Aboaid, Yin Mon Myat, Kyaw Zin Thein

**Affiliations:** 1 College of Osteopathic Medicine, Touro University, Henderson, NV 89014, USA; 2 Department of Internal Medicine, Kirk Kerkorian School of Medicine at UNLV, Las Vegas, NV 89106, USA; 3 Department of Internal Medicine, One Brooklyn Health—Interfaith Medical Center Campus, Brooklyn, NY 11213, USA; 4 Division of Hematology and Medical Oncology, Comprehensive Cancer Centers of Nevada, Central Valley, Las Vegas, NV 89169, USA

**Keywords:** Tissue-agnostic drugs, advances in cancer therapeutics, precision oncology updates

## Abstract

The ever-expanding development of tissue-agnostic therapies which target malignancies based on specific mutations rather than tissue origin have transformed the landscape of oncology. The purpose of this review is to explore the impact, safety, and challenges of tissue-agnostic therapies including pembrolizumab, dostarlimab, larotrectinib, entrectinib, repotrectinib, dabrafenib plus trametinib, selpercatinib, and trastuzumab deruxtecan. As the therapeutic arsenal continues to grow, it is crucial to understand how these therapies truly benefit patients and to address the barriers that stand in the way of making them more widely available. Although these therapies have shown effectiveness across multiple cancer types, substantial challenges persist, including overcoming the burden of intratumoral heterogeneity and resistance mechanisms that reduce therapeutic efficacy. We discuss emergence of pan-histological biomarkers, such as neoantigen burden, current updates on trials as well as trial outlining strategies to refining patient selection, while also supporting broader access to biomarker testing. Collectively, these insights underscore the transformative role of tissue-agnostic therapies in precision oncology while emphasizing the ongoing need for research to optimize their application and overcome current barriers.

## 1 Introduction

**Figure 1:**
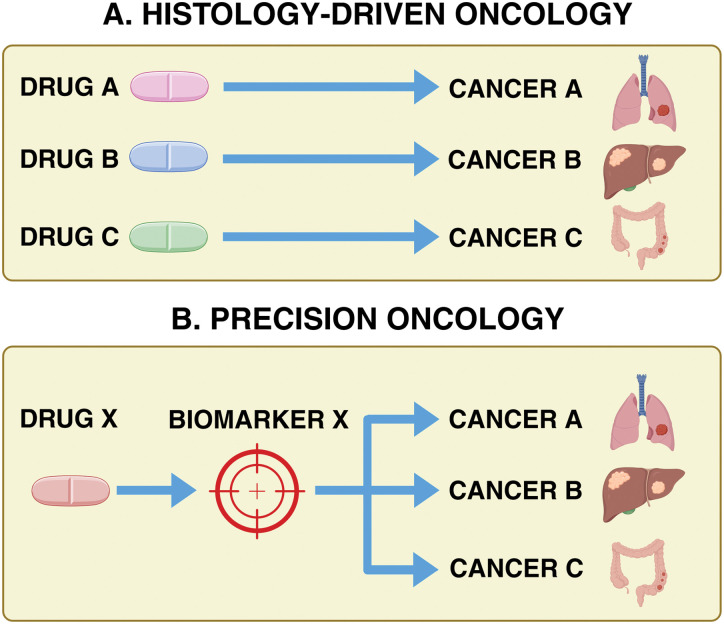
Histology-driven targeting of cancer therapeutics vs. tissue-agnostic targeting of cancer therapeutics (precision oncology). Historically, oncology treatments were guided by tumor histology. Tumor-agnostic therapies, however, target cancers based on shared biomarkers, such as mutations or fusions, regardless of tissue origin. Basket trials play a crucial role in evaluating their efficacy across diverse cancer types, expanding treatment options for patients, including those with rare malignancies who might not otherwise receive targeted therapies. Created in BioRender. Thein, K. (2025) https://BioRender.com/s80z131 (accessed on 18 August 2025)

Tissue-agnostic therapies represent a groundbreaking shift in oncology, marking a departure from the traditional approach of treating cancer based on its tissue of origin. Instead, these therapies focus on the molecular and genetic characteristics underlying the disease [1–3]. In the past, treatments were guided by the tumor’s location, which often left patients with rare or treatment-resistant cancers with limited to no options (Fig. 1). However, advances in genomics and precision medicine have changed this narrative. By targeting the genetic drivers shared across various cancers, these therapies address the mechanisms of tumor growth rather than their physical location, offering new hope for patients with complex and diverse cancer diagnoses [2,3].

This progress has been fueled by two main strategies: targeted therapies and immunotherapies. Targeted therapies are designed to inhibit specific molecular alterations, such as mutations, gene fusions, or overexpressed proteins, that are critical to tumor growth and survival [2–4]. For instance, drugs like larotrectinib and entrectinib block neurotrophic tyrosine kinase (*NTRK*) fusions, while dabrafenib and trametinib inhibit the mitogen-activated protein kinase (MAPK) pathway in cancers with *BRAF*^*V600E*^ mutations. By precisely targeting the genetic drivers of cancer, these treatments are often more effective and associated with fewer side effects compared to traditional chemotherapy [3].

Immunotherapies, on the other hand, harness the body’s immune system to combat cancer. Immune checkpoint inhibitors, such as pembrolizumab and dostarlimab, block pathways like Programmed Cell Death Protein-1/Programmed Cell Death Ligand-1 (PD-1/PD-L1) that tumors use to evade immune detection [5,6]. By reactivating T cells, these therapies enable the immune system to recognize and attack cancer cells. Immunotherapies have shown particular success in tumors with high immunogenicity, such as those characterized by microsatellite instability-high (*MSI-H*) or high tumor mutational burden [3].

Despite their differing mechanisms, these approaches share a common goal: improving patient outcomes by targeting cancer’s biology. Targeted therapies disrupt the pathways that fuel tumor growth, while immunotherapies restore the immune system’s ability to mount a defense. These advancements have led to the 2017 approval of pembrolizumab for *MSI-H* cancers marked a pivotal moment, signaling a shift toward biomarker-driven therapies rather than treatments based solely on anatomical classification [2,3,7].

## 2 Immunotherapy: Tissue-Agnostic Targeting of Cancer Therapeutics

### 2.1 MSI-H-Solid Tumors

The mismatch repair system utilizes several genes, including mutL homolog 1 (MLH1), mutL homolog 2 (MSH2), mutL homolog 6 (MSH6), and post-meiotic segregation increased 2 (PMS2) to repair errors made during DNA replication [6]. Defects in the mismatch repair (dMMR) system can lead to high mutation rates, known as DNA microsatellite instability. The continued inability to correct these mismatches leads to the introduction of more errors into the affected DNA, which may be used as potential targets for the development of new chemotherapies [8].

#### 2.1.1 Pembrolizumab in MSI-H Solid Tumors

**Figure 2:**
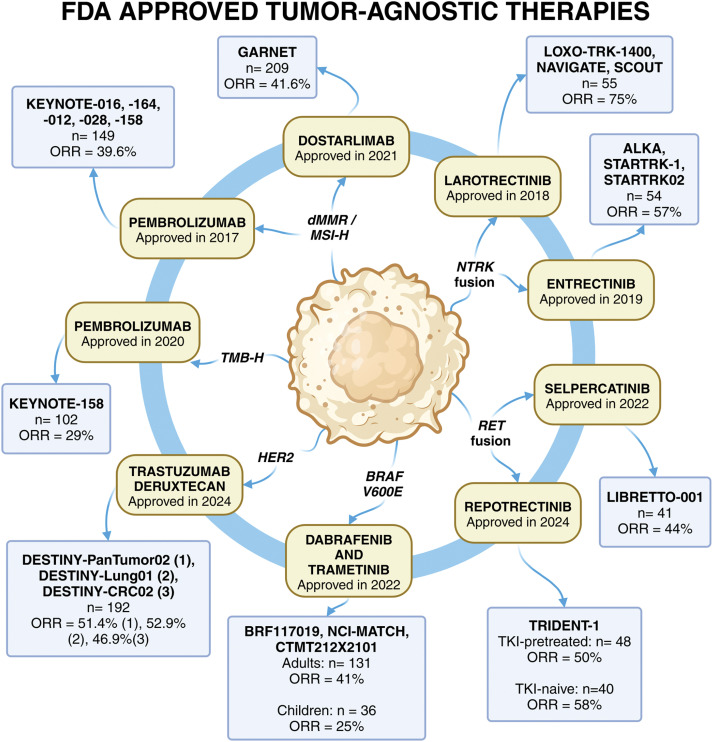
FDA approved tissue-agnostic targeting of cancer therapeutics. There are a total of eight FDA-approved tumor-agnostic therapies across nine different indications. The year of approval, as well as the clinical trials, the number of patients in the efficacy population and the ORR leading to the approval are summarized in the figure. These treatments include immunotherapy, such as the PD-L1 inhibitors, pembrolizumab and dostarlimab.Pembrolizumab was approved for both *dMMR*/*MSI-H* and *TMB-H* cancers, whereas dostarlimab was approved for *dMMR* cancers. Targeted therapies for oncogenic fusions include larotrectinib and entrectinib for *NTRK* fusions, as well as selpercatinib and repotrectinib for *RET* fusions. Dabrafenib (*BRAF* inhibitor) and trametinib (*MEK* inhibitor) represent the first combinational biomarker-driven treatment that was approved. Lastly, trastuzumab deruxtecan is an antibody-drug conjugate targeting *HER2*-positive solid tumors, which was approved based on three clinical trials. The continued development of tumor-agnostic therapies highlights the shift toward biomarker-driven precision oncology, expanding treatment options for patients across diverse cancer types, with more targeted therapies expected to emerge as research advances

Note: *BRAF*, v-raf murine sarcoma viral oncogene homolog B1; *dMMR*, deficient DNA mismatch repair; FDA, Food and Drug Administration; *HER2*, human epidermal growth factor receptor 2; *MEK*, mitogen-activated extracellular signal-regulated kinase; *MSI-H*, microsatellite instability-high; *NTRK*, neurotrophic tyrosine receptor kinase; n, number of patients; ORR, objective response rate; *RET*, rearranged during transfection; TKI, tyrosine kinase inhibitor; *TMB-H*, tumor mutational burden-high. Created in BioRender. Thein, K. (2025) https://BioRender.com/s80z131.

Pembrolizumab has transformed cancer treatment as the first FDA-approved therapy that works across multiple cancer types based on specific genetic markers, rather than the tumor’s location (Fig. 2). Nevertheless, pembrolizumab targets tumors with MSI-H or dMMR [7,8]. The primary endpoint of the KEYNOTE trials was objective response rate (ORR) assessed with RECIST criteria, and several secondary endpoints including duration of response (DOR), progression-free survival (PFS), and overall survival (OS) [7,8]. As depicted in Table 1, evidence from the pivotal KEYNOTE trials demonstrated its effectiveness, with an impressive objective response rate (ORR) of 39.6% across 15 different tumor types, such as colorectal, endometrial, and gastric cancers (Table 1). Even more striking, non-colorectal cancers showed a higher ORR of 46%, underscoring pembrolizumab’s broad potential. Additionally, the therapy achieved durable responses lasting over six months in 78% of patients, with some even experiencing complete remission [9].

**Table 1:** Summary of FDA-approved tissue-agnostic targeting of cancer therapeutics

Clinical trial (s)	Regimen	Biomarker target	Number of patients (n)	ORR (%)	FDA approval date
KEYNOTE-016 KEYNOTE-164	Pembrolizumab	*MSI-H* *dMMR*	149	39.6 (95% CI: 31.7–47.9)	23 May 2017
KEYNOTE-012					
KEYNOTE-028					
KEYNOTE-158					
LOXO-TRK-1400	Larotrectinib	*NTRK*	55	75 (95% CI: 61–85)	26 November 2018
NAVIGATE					
SCOUT					
ALKA	Entrectinib	*NTRK*	54	57 (95% CI: 43–71)	15 August 2019
STARTRK-1					
STARTRK02					
KEYNOTE-158	Pembrolizumab	*TMB-H* (≥10 mut/mb)	102	29 (95% CI: 21–39)	15 Jun 2020
GARNET	Dostarlimab	*dMMR*	209	41.6 (95% CI: 34.9–48.6)	17 August 2021
BRF117019 NCI-MATCH	Dabrafenib and trametinib	*BRAF* ^*V600E*^	Adults: 131 Pediatric: 36	Adults: 41 (95% CI: 33–50)	22 June 2022
CTMT212X2101				Pediatric: 25 (95% CI: 12–42)	
LIBRETTO-001	Selpercatinib	*RET*	41	44 (95% CI: 28–60)	21 September 2022
DESTINY- PanTumor02	Trastuzumab Deruxtecan	*HER2*	192	51.4 (95% CI: 41.7–61.0)	05 April 2024
DESTINY-Lung01 DESTINY-CRC02				52.9 (95% CI: 27.8–77.0)	
				46.9 (95% CI: 34.3–59.8)	
TRIDENT-1	Repotrectinib	*NTRK*	88 (TKI- pretreated: 48, TKI-naive: 40)	TKI- pretreated: 50 (95% CI: 35–65) KI-naive: 58 (95% CI: 41–73)	13 June 2024

Note: *BRAF*, v-raf murine sarcoma viral oncogene homolog B1; *dMMR*, deficient DNA mismatch repair; FDA, Food and Drug Administration; *HER2*, human epidermal growth factor receptor 2; *MSI-H*, microsatellite instability-high; mut/mb, mutations per megabase; *NTRK*, neurotrophic tyrosine receptor kinase; n, number of patients; ORR, objective response rate; *RET*, rearranged during transfection; TKI, tyrosine kinase inhibitor; *TMB-H*, tumor mutational burden-high; CI, confidence interval.

Pembrolizumab works by blocking the PD-1 receptor on T cells, preventing it from interacting with its ligands PD-L1 and PD-L2 [6,9]. This mechanism, known as immune checkpoint blockade, reinvigorates the immune system’s ability to recognize and attack cancer cells, a hallmark of immunotherapy [6,9,10]. While its benefits are well-documented, pembrolizumab is not without challenges. Immune-related adverse events (irAEs) like colitis, pneumonitis, and endocrinopathies can occur, but they are typically manageable with timely treatment [11–13].

Despite its remarkable impact, pembrolizumab has some limitations. It has shown limited effectiveness in primary brain tumors due to the distinct tumor environment and genetic complexity [14]. Additionally, the high cost of genomic testing for MSI-H status can make access difficult for some patients [15]. Nevertheless, pembrolizumab represents a major milestone in oncology, showcasing the promise of immunotherapy in treating cancers defined by specific biomarkers [6,7].

#### 2.1.2 Dostarlimab in MSI-H-Solid Tumors

Dostarlimab is an IgG4 monoclonal antibody that functions to target and inhibit the PD-1/PD-L1 pathway similarly to pembrolizumab [16,17]. Dostarlimab has made significant strides in cancer treatment, particularly for patients with tumors characterized by MSI-H or dMMR. The KEYNOTE-158 and PHAEDRA studies yielded promising results in patients with endometrial cancer (EC) with the use of monoclonal antibodies [16]. The data from these studies lead to the approval of dostarlimab by the FDA in 2021 for its remarkable promise in advanced or recurrent endometrial cancer, offering a much-needed option for patients with limited alternatives [16–18].

The GARNET trial highlighted the drug’s efficacy in phases and cohorts of solid neoplasms, highlighting its utility in not only treating advanced/recurrent EC, but also colorectal and other solid tumor types. The overall ORR among all cohorts seen by this trial was 41.6 (95% Confidence Interval (CI): 34.9–48.6) [16]. The response rates are comparable to those observed with pembrolizumab, and in some cases, patients have achieved prolonged remission. Its success in refractory cases, where other treatments have failed, underscores dostarlimab’s potential to address unmet needs in oncology. However, most of the available data focuses on endometrial cancer, and while early findings suggest it may be effective against a range of tumor types, further studies are needed to confirm its broader applicability [16]. Additional ongoing research has revealed the ability to replace chemotherapy, radiation and surgery, in patients with rectal cancer with ICIs, to preserve organ function and maintain quality of life by avoiding permanent -ostomy creation [16].

The safety profile of dostarlimab has been another focus of clinical research. irAEs, such as colitis and dermatitis, are relatively common but generally mild and manageable with timely care [16,18]. These side effects stem from the drug’s mechanism of action, which activates the immune system and can inadvertently lead to inflammation in healthy tissues [16]. Despite these challenges, most patients tolerate the drug well when irAEs are managed appropriately, allowing them to continue treatment and benefit from its therapeutic effects.

Dostarlimab’s approval has opened new doors in cancer therapy, but it also comes with ongoing discussions. Its use beyond endometrial cancer remains an area of active research, as data on other tumor types is still limited. Comparisons with pembrolizumab, which has more extensive clinical evidence, also raise questions about how best to integrate dostarlimab into existing treatment frameworks. Additionally, the financial barriers associated with immune checkpoint inhibitors and the cost of genomic testing to identify MSI-H or dMMR status present challenges for broader access [16,17].

Despite these considerations, dostarlimab has already proven to be a valuable addition to the immunotherapy toolkit, offering hope to patients with MSI-H or dMMR cancers. Its ability to deliver durable responses and manage refractory cases highlights its potential to improve outcomes for many patients, even as research continues to expand its applications.

#### 2.1.3 Resistance Mechanisms in MSI-H Cancers

Mechanisms of resistance for MSI-H tumors involve the activation and upregulation of alternative signaling pathways that allow for bypassing the effects of immune checkpoint inhibitors (ICIs) [19]. Mutations in the janus kinases 1 and 2 have led to the inactivation of the JAK-STAT pathway which directly decreases a cell’s ability to induce proteasomes and decreasing MHC-I antigen presentation while increasing tumor cell survivability [20]. This resistance can lead to clonal evolution and the further emergence of subpopulations that are less responsive to ICIs [20]. Other means of altering the efficacy of ICIs have been thought to be based on intratumoral microenvironment, epigenetic modifications, and dysbiosis of the gut microbiome [20]. Patients with a higher diversity of the gut microbiome, specifically with *Faecalibacterium* and *Ruminococaceae*, have enhanced antigen presentation and anti-tumoral immune responses, whereas patients with an abundance of *Bacteriodales*, demonstrated a decreased immune response [21]. Ongoing research into these mechanisms may provide further insight into the development of a range of therapeutic options for treating *MSI-H* cancers.

### 2.2 Tumor Mutational Burden-High (TMB-H) Solid Cancers

Tumor mutational burden (TMB) refers to the number of somatic mutations per megabase of coding DNA. TMB-H was previously defined as having ≥175 mutations per exome [22]. Since the FDA approval of the FoundationOne CDx genomic profiling assay, which demonstrated the ability to identify patients with TMB-H solid cancers as ≥10 mutations/megabase that may benefit from immunotherapy such as pembrolizumab [23]. These high rates of mutations lead to the formation of neoantigens, which prompt a stronger immune response [24]. This mutational burden can act as a biomarker for the development of immune checkpoint inhibitors [24,25].

#### 2.2.1 Pembrolizumab in TMB-H Solid Cancers

Pembrolizumab’s approval for treating cancers with TMB-H has broadened its role in oncology, offering a critical option for patients with challenging tumor profiles [1,26,27]. The abundance of neoantigens makes TMB-H tumors particularly responsive to immunotherapies like pembrolizumab. This mechanism has been instrumental in delivering durable responses for patients across multiple tumor types, even in cases where other treatments have failed [26,27].

Clinical trials, most notably KEYNOTE-158, have demonstrated pembrolizumab’s effectiveness in TMB-H cancers, showing significant and lasting responses in a variety of tumors with an ORR of 29% (95% CI: 21–39) [5], (Table 1). This led to another approved indication by the FDA for treating TMB-H solid cancers in patients who did not have other alternative treatment options [26,27]. These results highlight pembrolizumab’s broad applicability, providing new hope for patients with cancers that were previously difficult to treat. However, while its efficacy has been impressive, the question of who stands to benefit the most remains a topic of ongoing research. Determining the best threshold for defining TMB-H has been challenging, with different studies and real-world practices often using inconsistent cutoffs [28]. Standardizing these criteria is essential to ensure that patients most likely to benefit from pembrolizumab can be accurately identified [28]. In terms of safety, pembrolizumab’s use in TMB-H cancers is consistent with its established profile in MSI-H tumors [11–13]. The drug’s overall tolerability in clinical trials and real-world settings has provided reassurance, making it a viable option for many patients [5].

Despite these advancements, discussions about pembrolizumab’s role in TMB-H cancers continue. The confounding biology of *MSI-H* status with *TMB-H* status in colorectal cancer has demonstrated that *MSI-H* status is the primary determinant of benefit from immunotherapy [29]. The variability in defining TMB-H poses challenges not only for clinical trials but also for ensuring equitable access to treatment [30]. The high cost of genomic testing needed to identify TMB-H status can limit access for some patients, further complicating its implementation [30]. Addressing these issues is critical for maximizing the drug’s potential and ensuring that it reaches all eligible patients [30].

Pembrolizumab’s approval for TMB-H cancers has marked a significant milestone in precision medicine. Its ability to deliver durable responses across a range of tumors reaffirms its importance in cancer immunotherapy. While refining patient selection and addressing barriers to access remain areas for improvement, pembrolizumab has already made a profound impact on the lives of many patients with biomarker-driven cancers.

#### 2.2.2 Resistance Mechanisms in TMB-H Cancers

TMB-H cancers have also been shown to have several means of resisting therapies. One mechanism of resistance has been shown to reduce the expression of antigen-presenting major histocompatibility complex class I molecules, leading to the failure of the immune system to recognize those cancer cells and deficiencies in antigen processing machinery [24,25,31]. Additionally, intratumor heterogeneity contributes to the diversity within tumor profiles themselves, which may result in the emergence of resistant clones that can survive and proliferate [32].

## 3 Targeted Therapy: Tissue-Agnostic Targeting of Cancer Therapeutics

### 3.1 NTRK-Fusion Positive Solid Cancers

The *NTRK* genes code for the tropomyosin receptor kinase (TRK) receptor proteins. *NTRK1*, *NTRK2*, and *NTRK3* code for *TRK-A*, *TRK-B*, and *TRK-C*, which activate the phosphoinositide 3-kinase (*PI3K*), rat sarcoma viral oncogene homolog/mitogen-activated protein kinase (*RAS/MAPK*), and phospholipase C (*PLC*) pathways, respectively [33]. These cellular pathways play a vital role in overall cell growth, proliferation, and survival. *NTRK* fusions are chromosomal rearrangements that are highly prevalent in rare malignancies, such as thyroid cancer, salivary gland cancer, and mammary analogue secretory carcinoma [33,34]. In contrast, NTRK gene fusions occur in less than 5% of common cancers, including pulmonary carcinoma, colorectal carcinoma, and cutaneous melanoma [35]. This molecular targeting has proved useful and effective in treating diverse tumor types [34].

#### 3.1.1 Larotrectinib in NTRK-Fusion Positive Solid Cancers

Laroctretinib is a first-generation TRK inhibitor approved in 2018 to treat advanced *NTRK* fusion-positive solid tumors in both adults and children [36–38]. Laroctretinib works by competitively inhibiting the ATP-binding site on the TRK receptor intracellular kinase domain and subsequently disrupting the tumor [39].

To assess the clinical efficacy of larotrectinib, multiple studies have been conducted to examine its impact on *NTRK* fusion-positive tumors. A pooled analysis of clinical trials LOXO-TRK-14001, SCOUT, and NAVIGATE demonstrated that larotrectinib effectively treated *NTRK-*positive tumors, with ORR as the primary endpoint [40]. LOXO-TRK-14001 included patients with advanced solid tumors, SCOUT included pediatric patients with advanced solid tumors, and NAVIGATE included patients with *NTRK* fusion-positive tumors [41,42]. The most common cancer types treated were salivary gland cancer, soft tissue carcinoma, infantile fibrosarcoma, and thyroid cancer [43]. This initial pooled analysis yielded an ORR of 75% (95% CI: 61–85) with a median duration of response (mDOR) of 35.2 months, while an updated pooled analysis produced an ORR of 66% with a median progression-free survival time of 46.2 months [36,43]. Additionally, a post hoc analysis including patients with brain metastases revealed an ORR of 68% and an mDOR of 48.7 months [43]. This pooled data not only reinforces larotrectinib’s clinical utility but also provides detailed insights into its optimal therapeutic impact.

While larotrectinib has shown diverse clinical applications, it is also important to evaluate its safety profile and tolerability. The most common adverse events include elevated liver enzymes, anemia, neutropenia, cough, constipation, diarrhea, and nausea. Because larotrectinib metabolism occurs primarily via hepatic cytochrome P450 3A4, drug-drug interactions must be carefully considered when dosing [39]. These adverse effects and drug interactions require careful monitoring and further investigation, so that patients can safely reap the therapeutic effects [39].

*NTRK* fusions have been detected with various testing methods, including fluorescence *in situ* hybridization (FISH), immunohistochemistry, and next-generation sequencing. Due to poor mapping quality and long intronic regions found in the NTRK genes, DNA-based next-generation sequencing does not yield reliable results for detection [44]. Additional efforts have been to circumvent this with the use of RNA-based next-generation sequencing, but has also shown to be limiting due to the labile nature of RNA [44,45]. However, more analysis must be performed on which method most optimally balances cost, accuracy, and turnaround time. Furthermore, the number of possible *NTRK* fusion partners may be infinite, which may complicate *NTRK* detection. Reliable and standardized diagnostic methods will expand patient accessibility and TRK inhibitor applicability to cancer treatment [44].

Larotrectinib provides effective treatment and durable response for numerous *NTRK*-fusion positive tumors. With continued adverse effect evaluation and evolving *NTRK*-fusion detection, larotrectinib stands as a groundbreaking option in precision oncology.

#### 3.1.2 Entrectinib in NTRK-Fusion Positive Solid Cancers

Shortly after larotrectinib, another first-generation TRK inhibitor known as entrectinib was approved by the FDA in 2019 [46]. Entrectinib uses the metabolite M5 to competitively inhibit *NTRK*, ROS proto-oncogene 1, tyrosine kinase (*ROS1*), and anaplastic lymphoma kinase (*ALK*) [47]. Entrectinib uniquely remains in the central nervous system as a weak p-glycoprotein substrate and demonstrates weak interaction with efflux transporters, which allows for better blood-brain barrier penetration than larotrectinib [48]. Because of its versatile multikinase properties, entrectinib is approved both for the treatment of advanced or metastatic *NTRK* fusion-positive tumors in adult and pediatric patients with disease progression following treatment or without satisfactory alternative therapy and for the treatment of *ROS1*-positive non-small cell lung cancer (NSCLC) [49,50]. These approvals are rooted in the numerous clinical trials exploring entrectinib’s efficacy across different tumor types [49,50].

Extensive analyses have evaluated entrectinib’s therapeutic impact. ALKA-371-001, STARTRK-1, and STARTRK-2 each included patients with advanced or metastatic *NTRK* fusion-positive tumors with ORR as the primary endpoint [47,51]. The most common cancer types included sarcoma, NSCLC, mammary analogue secretory carcinoma, breast cancer, thyroid cancer, and colorectal cancer. A pooled analysis of these three trials demonstrated an ORR of 57.4% (95% CI: 43.2–70.8) with a mDOR of 10.4 months (95% CI: 7.1–Not Estimable (NE)), while an updated pooled analysis showed an ORR of 61.2% (95% CI: 51.9–69.9) and mDOR of 20 months (95% CI: 13–38.2) [47,51]. An analysis of patients with brain metastasis yielded an intracranial ORR of 63.6% [45]. Additionally, a pooled analysis of the STARTRK-NG and phase II TAPISTRY trials demonstrated efficacy in pediatric patients with an ORR 70% (95% CI: 51–84) and mDOR of 25.4 months (95% CI: 14.3–NE) [46]. These promising results have established entrectinib as a reliable choice in cancer treatment.

As with most targeted therapies, entrectinib administration has specific safety concerns. A variety of adverse effects may be expected, including dizziness, diarrhea, constipation, cough, and weight gain [47]. More serious adverse events include QT interval prolongation, vision changes, and neurotoxicity [47]. Additionally, entrectinib metabolism by cytochrome P450 3A4 and CA5 indicates potential drug-drug interactions that must be accounted for when dosing. Further research must be conducted to fully characterize these safety concerns and their long-term implications [49].

Entrectinib has proven itself an important multikinase inhibitor with durable response against *ROS1*-positive NSCLC and NTRK fusion-positive tumors with CNS metastatic potential. If balanced with potential adverse effects, entrectinib’s versatility allows it to optimize patient outcomes.

#### 3.1.3 Repotrectinib in NTRK-Fusion Positive Solid Cancers

As tumors developed resistance to first-generation TRK inhibitors, second-generation TRK inhibitors, such as repotrectinib, were created. Repotrectinib is a small-molecule kinase inhibitor that specifically targets several tropomyosin receptor kinases including *ROS1*, *TRKA*, *TRKB*, and *TRKC* [52–54]. Repotrectinib was approved in 2023 for locally advanced or metastatic *ROS1*-positive NSCLC and in 2024 for advanced or metastatic *NTRK* fusion-positive solid tumors in adults and pediatric patients 12 years and older [55]. These recent approvals were supported by clinical trials demonstrating repotrectinib’s unique tumor applications.

The primary clinical trial for repotrectinib, TRIDENT-1, provided critical evidence supporting repotrectinib’s clinical benefits. TRIDENT-1 included patients with advanced solid tumors expressing *ALK*, *ROS*, and *NTRK* rearrangements. Akin to the trials that led to the approval of first-generation TRK inhibitors, the primary endpoint of TRIDENT-1 was ORR. This study yielded an ORR of 58% in tyrosine kinase inhibitor (TKI)-naive patients and an ORR of 50% in TKI-pretreated patients. Additionally, a response was detected in each of the 5 patients with CNS metastatic disease [56–58]. Repotrectinib provides an effective second-generation treatment option for advanced tumors that may not be treatable by other means.

Repotrectinib has proven effective in treating rare tumors, but not without notable side effects. Adverse effects of repotrectinib include dizziness, nausea, constipation, peripheral neuropathy, ataxia, cognitive impairment, dyspnea, muscular weakness, and fatigue [59]. Because of repotrectinib’s novelty, more research is needed to balance efficacy with tolerability.

#### 3.1.4 Resistance Mechanisms in NTRK-Fusion Positive Solid Tumors

The approval of NTRK inhibitors has revolutionized cancer therapy but also opened many discussions. As tumors progress, they may acquire resistance to TRK inhibitors. Repotrectinib has shown efficacy in targeting tumors with solvent front mutations (SFM), *ALK*, and other non-SFM mutations. Although no specific NTRK fusion gene partners have been found resistant to therapies, mechanisms of resistance include *NTRK* solvent front mutations, *xDFG* motif, gatekeeper residue mutations, which directly affect drug binding [60]. Additionally, tumors may utilize alternative signaling pathways or downstream pathway mutations, allowing the tumor to bypass the effects of NTRK inhibition [61,62]. Meanwhile, other second-generation TRK inhibitors, such as selitrectinib and taletrectinib, are currently under development. Combination therapy, such as selitrectinib with crizotinib, may also prove useful to bypass resistance mechanisms [63]. However, the rarity of *NTRK* fusions poses significant challenges to the continued evaluation of new therapies [33,47]. Although further research and collaborative efforts are necessary to address these limitations, repotrectinib has already secured itself as a cornerstone therapy supported by promising clinical results.

### 3.2 V-Raf Murine Sarcoma Viral Oncogene Homolog B (BRAF ^***V600E***^)-Altered Solid Cancers

#### 3.2.1 Dabrafenib Plus Trametinib in BRAF^*V600E*^-Altered Solid Cancers

*BRAF* Class I mutations possess strong kinase activity compared to *BRAF* Class II and Class III and are solely found at the V600 locus, making this mutation class a target for new cancer therapies [64]. The combination therapy with dabrafenib, a *BRAF* inhibitor, and trametinib, a mitogen-activated protein kinase kinase (*MEK*) inhibitor, targets the *MAPK* signaling cascade for phosphorylating several proteins, including *RAS*, *BRAF*, *MEK*, and extracellular signal-regulated kinase (*ERK*) [65,66]. In combination, this therapy regulates several processes in the tumor life cycle, including cell proliferation, survival, and differentiation in cancers with *BRAF*^*V600E*^ mutations. Blocking the BRAF protein and inhibiting *MEK* in combination effectively disrupts the activation of the *MAPK* pathway [65–67]. This combination therapy was approved in June 2022 for the treatment of unresectable or metastatic solid tumors, mainly biliary tree cancer (BTC), high-grade glioma (HGG), and low-grade glioma (LGG), which exhibited *BRAF*^*V600E*^ mutations in adult or pediatric patients ≥6 years of age [65–67].

One of the greatest upsides to using dabrafenib is that it targets mutations in rare cancer types, including gliomas, biliary tract cancer, and gastrointestinal stromal tumors, among others [68]. An important drawback to using dabrafenib monotherapy is the paradoxical activation of the *MAPK* pathway, hence why trametinib is utilized in combination for disrupting the *MAPK* downstream proteins *MEK1* and *MEK2* [68].

Three trials (ROAR, NCI-MATCH, and Study X2101) evaluated the effectiveness of dabrafenib and trametinib focused on treating various cancer types exhibiting *BRAF*^*V600E*^ mutations with the primary endpoint being ORR; ROAR and NCI-MATCH (adult patient population), and Study X2101 trials (pediatric patient population as young as six years old) [65,68,69]. The adult populations demonstrated an ORR of 46% in biliary tract cancer (95% CI: 31–61), 33% for high-grade glioma (95% CI: 20–48), and 50% for low-grade glioma (95% CI: 23–77) [65,68,69]. Comparably, Study X2101 demonstrated an ORR was 25% (95% CI: 12–42) and a duration of response (DOR) of ≥6 months for 78% of patients and ≥24 months for 44% of patients [65].

Among the adult populations, the most frequent adverse effects with this combination therapy included pyrexia, headache, hemorrhage, fatigue, rash, nausea, vomiting, diarrhea, constipation, cough, myalgia, arthralgia, and edema [65]. The pediatric population in Study X2101 exhibited similar adverse effects with the addition of dry skin, dermatitis, acneiform, abdominal pain, and paronychia [68].

#### 3.2.2 Resistance Mechanisms in BRAF^*V600E*^-Altered Solid Cancers

One mechanism of resistance that the *BRAF*^*V600E*^ mutation utilizes is driven by alterations in MAPK-dependent and MAPK independent pathways, thereby evading selective inhibition [66]. Other alterations in these signaling pathways involve the hyperactivation of tyrosine kinases seen in the *PI3K/AKT* pathway [70,71]. This leads to secondary mutations that alter downstream pathways to circumvent inhibition, seen with mutations in components including *NRAS* [70,71]. Although proven effective for inhibiting the *MAPK* signaling cascade in cancers with *BRAF*^*V600E*^ mutations, further research is necessary to improve the current effectiveness of dabrafenib + trametinib combination therapy while also limiting the common adverse effects.

### 3.3 Rearranged during Transfection (RET)-Altered Solid Cancers

Rearranged during transfection (*RET*) is a transforming proto-oncogene which encodes a receptor tyrosine kinase initially discovered in a patient with T-cell lymphoma [72]. Since its discovery, it has been shown to be involved in the fetal development of several body systems, including the nervous, genitourinary, hematopoietic, and gastrointestinal systems [73–75]. Due to its involvement in the embryogenesis of several body systems, RET fusions and mutations are found in a diverse set of cancers including lung, colorectal, breast, and thyroid cancers [76,77]. Activation of this oncogenic driver is done mainly through gene fusions and point mutations with >35 different *RET* fusion genes, more commonly seen with NSCLC and papillary thyroid cancer (PTC) [78–80]. In contrast to activation by fusion mutations seen in NSCLC and PTC cancers, multiple endocrine neoplasia type 2 (MEN2) syndromes display *RET* point mutations that cluster in the cysteine-rich domain in exons 10, 11, and the tyrosine kinase domains in exons 13 and 16 [81,82]. The *RET*^*M918T*^ point mutation is the most common and most aggressive within the kinase domain of the *RET* gene [83].

#### 3.3.1 Selpercatinib in RET-Altered Solid Cancers

Selpercatinib is a tyrosine kinase inhibitor directed towards inhibiting the rearranged during transfection (*RET*) gene, a proto-oncogenic transmembrane receptor kinase [84,85]. Selpercatinib functions to inhibit downstream signaling cascades, further disrupting important processes for tumor growth and proliferation. Selpercatinib’s expedited approval in 2020 has provided another treatment option for patients with metastatic *RET* fusion-positive NSCLC and metastatic *RET* mutant medullary thyroid cancers [84,85]. It received further FDA approval for its utilization in unresectable or metastatic *RET*-positive cancers, locally advanced or metastatic solid tumors in patients ≥ 12 years old, in September 2022 [84,85].

Early efficacy data in the LIBRETTO-001 phase I/II recommended dosing at 120 mg twice daily (<50 kg) or 160 mg twice daily (≥50 kg) [86]. Further evaluation of the efficacy of selpercatinib was done in an ongoing multicenter, open-label multi-cohort trial in 41 patients with *RET* fusion-positive tumors beyond NSCLC and thyroid cancers. These tumor types included pancreatic adenocarcinoma, colorectal, salivary, unknown primary, breast, soft tissue sarcoma, bronchial carcinoid, ovarian, small intestine, and cholangiocarcinoma [87]. The ORR was 44% (95% CI: 41–87) with a DOR of 24.5 months (95% CI: 9.2–NE). At 6 months, 67% (95% CI: 41–87) were continuing to respond positively to selpercatinib, and 56% at 12 months (95% CI: 31–78) [87].

Although selpercatinib has shown incredible benefit to many cancer types, patients in these early trials experienced a range of adverse reactions including nausea, dry mouth, fatigue, edema and rash; more serious adverse effects being hypertension and elevated liver aminotransferases [86,87].

Recent updates to LIBRETTO-001 have included 52 more patients and 16 months of longer follow-up. Most patients in this update had GI *RET*-positive cancers (n = 31), with pancreatic and colorectal being the most common. Among this group of patients, the ORR was 44.2% (95% CI: 30.5–58.7), with pancreatic tumors being 53.8% and 30.8% in colorectal tumors [88]. The median DOR among this group across all tumor types was 37.2 months [88]. A significant number of colorectal cancer (CRC) patients also had *MSI-H* status, suggesting that combinational therapies may provide further benefit [88]. This updated data further demonstrates the vast anti-tumor activity against *RET*-positive tumors and further provides hope in impacting the lives of patients with several types of solid-organ, metastatic, *RET*-positive cancers.

#### 3.3.2 Resistance Mechanisms in RET-Altered Solid Cancers

*RET*-altered cancers utilize gatekeeper mutants as a mechanism of resistance to treatments, as seen in *RET*^*V804M/L*^ [89–91]. These mutations interrupt the ability to bind to the *RET* kinase domains by creating steric hindrance and preventing binding due to the *V804* gatekeeper and invariant gatewall *K758* residues [89–91]. Solvent front mutations (SFMs) are variations in the surface-exposed amino acid sequence which exist in close proximity to the ATP-binding site in kinases, causing electrostatic forces and reorientation of the surrounding residues [92]. These *RET*^*G810*^ SFMs demonstrated the first described recurrent mechanism by which tumor cells have shown to be resistant against selpercatinib, emerging as early as 3 months after starting treatment [93]. Further research directed at inhibiting gatekeeper mutations, while maintaining activity against SFMs can prove to be the key in treating *RET* resistance [93].

## 4 Antibody Drug Conjugate: Tissue-Agnostic Targeting of Cancer Therapeutics

### HER2-Positive Solid Cancers

#### Trastuzumab Deruxtecan in HER2-Positive Solid Cancers

Trastuzumab deruxtecan has brought a new wave of optimism to the treatment of *HER2*-positive solid tumors. This innovative therapy combines the precision of *HER2*-targeting trastuzumab with a powerful chemotherapy payload, deruxtecan, delivered directly to cancer cells expressing *HER2* receptors [94–96]. The result is a targeted yet potent attack on tumors, sparing healthy cells and minimizing many of the broader side effects seen with traditional chemotherapies; a remarkable step forward for patients who often face limited options when their cancer progresses.

In the DESTINY-PanTumor02 trials, trastuzumab-deruxtecan (T-DXd) demonstrated exceptional efficacy, particularly in patients with advanced or metastatic *HER2*-positive breast cancer. Among those who had undergone extensive prior treatments, this therapy achieved an ORR of 51.4% (95% CI: 41.7–61.0) and a mDOR of 19.4 months, offering many patients durable and meaningful responses [94,95]. Importantly, this therapy has shown promise beyond breast cancer, extending to *HER2*-positive gastric and non-small cell lung cancers [95].

However, as with any powerful treatment, there are risks. One of the more serious concerns with trastuzumab deruxtecan is interstitial lung disease (ILD), which, although rare, requires close monitoring and swift intervention if symptoms develop [95,96]. Other side effects, such as nausea, fatigue, and reduced blood cell counts, are more common but generally manageable with supportive care [94–96]. Despite these challenges, the benefits of the therapy often outweigh the risks for patients who have run out of options [96].

The FDA’s approval of T-DXd for *HER2*-positive solid cancers represents a significant shift in how we think about *HER2*-driven diseases. While issues like its high cost (as much as $4281/cycle) remain, ongoing research is looking to refine its use and extend its reach to other tumor types [97]. For patients and their families, trastuzumab deruxtecan offers hope where little existed before—a testament to the power of combining cutting-edge science with compassionate care.

#### Resistance Mechanisms in HER2-Positive Solid Cancers

Several mechanisms of resistance have been seen in the use of current treatments for *HER2*-positive solid cancers including mutations within the *HER2* receptor leading to alterations in structure, thereby reducing the ability for therapies to bind and inhibit tumor development [98]. Epigenetic alterations have also shown to play a role in resistance, allowing for tumor cells to rely on constitutive activation of parallel signaling pathways involving MET proto-oncogene receptor tyrosine kinase (*MET*), Human Epidermal growth factor Receptor 3 (*HER3*) and fibroblast growth factor receptor (*FGFR*) to bypass *HER2* inhibition [98,99]. The involvement and activation of the cyclin D1-CDK4/6 proteins that regulate the G1-S transition in the cell cycle have also been implicated in the resistance of treatment, enhancing the ability to escape from antibody-dependent cellular cytotoxicity [98–100].

## 5 Challenges and Future Directions

Tissue-agnostic therapies, while groundbreaking, face significant challenges that limit their potential. One of the key obstacles is the development of resistance. Tumors often adapt to treatment by acquiring secondary mutations, reactivating alternative signaling pathways, or evading immune system detection—all of which are hallmarks of cancer—which can undermine the effectiveness of these therapies over time [101]. To overcome these resistance mechanisms, researchers are exploring innovative solutions, including combination therapies that target multiple pathways simultaneously, aiming to improve treatment durability and outcomes.

Another major hurdle is access to the advanced diagnostics required to identify patients who could benefit from these therapies. Molecular testing, essential for detecting biomarkers like *MSI-H* or *TMB-H*, can be prohibitively expensive and is often unavailable in resource-limited regions [16,17,30]. This disparity creates significant barriers to equitable treatment access. Addressing this issue will require expanding diagnostic infrastructure, lowering the costs of testing, and ensuring that these advancements are accessible to patients everywhere.

Adaptive clinical trial designs, such as basket trials, have shown promise in evaluating tissue-agnostic therapies across diverse tumor types. These trials allow researchers to test treatments based on genetic markers rather than tumor location, accelerating the development of new therapies. However, they face challenges, including small sample sizes and the variability of patient populations, which can make it difficult to draw definitive conclusions [102]. To maximize their effectiveness, these trials require careful design and collaboration among researchers, clinicians, and regulatory agencies.

Despite these obstacles, tissue-agnostic therapies represent a significant leap forward in precision medicine. By addressing resistance, improving access to diagnostics, and refining clinical trial methods, the field can continue to advance and bring these transformative treatments to more patients in need [2]. Adaptive clinical trial designs will require collaboration among clinicians, governmental regulatory agencies, and the pharmaceutical industry in order to continue to bring promise and hope for patients and their families.

## 6 Future Tissue-Agnostic Targeting of Cancer Therapeutics

Advancements in precision oncology continue to expand as more tumor-agnostic treatments are approved. *KRAS*^*G12C*^ inhibitors, fibroblast growth factor receptor (FGFR) inhibitors, and neuregulin 1 (NRG1) fusion inhibitors show promise across multiple cancers, with ongoing trials evaluating their efficacy.

Kirsten rat sarcoma viral oncogene homolog (*KRAS*) serves as a prominent oncogenic target due to its prevalence in human cancers. Despite its early discovery, researchers initially considered it undruggable [103]. The identification of the switch II pocket in the *KRAS*^*G12C*^ isotype enabled targeted therapies to bind the site, yielding promising results for pan-cancer populations, especially in patients with NSCLC [103]. Covalent *KRAS*^*G12C*^ inhibitors, such as sotorasib and adagrasib, have shown success in the CodeBreaK and KRYSTAL trials, respectively. In a pan-cancer cohort in the KRYSTAL-1 trial, 64 patients who received adagrasib had an ORR of 35.1%, mDOR of 5.3 months, and median progression-free survival (mPFS) of 7.4 months [104]. Sotorasib demonstrated its potential as a tumor-agnostic agent in the CodeBreaK 100 trial, with 59 patients with NSCLC showing an ORR of 32.2%, and 42 patients with CRC showing an ORR of 7.1% [105]. Divarasib is another *KRAS*^*G12C*^ inhibitor that exhibited an ORR of 53.1% in NSCLC and 29.1% in CRC patients [106]. Although cross-trial data are limited, divarasib demonstrates numerically superior efficacy compared to sotorasib and adagrasib. The study included tumor types beyond NSCLC and CRC, but was constrained by the patient population. Variations in efficacy across cancers may stem from biological differences, the distribution of mutations, and resistance mechanisms. For example, *KRAS*^*G12D*^ inhibitors are under investigation but face challenges due to the absence of a cysteine residue adjacent to the switch II pocket, thus, a new approach was required to access the binding site. Additionally, the *KRAS*^*G12D*^ mutation is more prevalent in cancers like pancreatic ductal adenocarcinoma (PDAC), distinguishing it from *KRAS*^*G12C*^, which occurs in other tumor types [107]. A Phase 1 trial using zoldonrasib (RMC-9805) is currently being studied as a promising treatment for patients with the *KRAS*^*G12D*^ mutation, and works as a molecular-glue to target the constitutively-activated *KRAS* protein [108].

*FGFR* is a family of tyrosine kinases involved in cellular proliferation and survival. The RAGNAR trial demonstrated that erdafitinib achieved an ORR of 64% in patients with *FGFR*1-3 alterations (mutations and fusions) across 16 solid tumor types, outperforming other FGFR inhibitors with lower response rates [109]. In contrast, the NCI-MATCH trial reported a more modest ORR of 16% among 25 patients with *FGFR* alterations, with a median duration of response of less than six months. Erdafitinib’s toxicity profile may restrict its pan-cancer use, as it showed higher rates of dose reduction (63%), interruption (75%), and discontinuation (10%) compared to other FGFR inhibitors [110]. Comparable to the RAGNAR trial, the phase 2 trial, FIGHT-207, examined the tumor-agnostic capabilities of pemigatinib as a *FGFR1-FGFR3* inhibitor, in three cohorts (Cohort A: *FGFR* fusions/rearrangements, Cohort B: *FGFR* actionable single nucleotide variants (SNVs), and Cohort C: *FGFR* kinase domain mutations and variants of uncertain significance) [111]. The ORR in cohort A, B, and C was 26.5%, 9.4%, and 3.8%, respectively [111]. Further investigation efforts are necessary to examine the challenges in targeting *FGFR* alterations including mutation diversity, diagnostic limitations, and tumor heterogeneity.

*NRG1* is a ligand that binds to *HER* receptors. *NRG1* fusions result in overexpression of the NRG1 protein, which can promote oncogenesis via upregulation of proliferative pathways [112]. The recent development of zenocutuzumab represents a unique mechanism of action as a bispecific antibody. It targets *NRG1* fusions by binding to both *HER2* and *HER3* on the surface of cancer and immune cells [113]. This interaction brings cancer cells closer to immune cells, promoting antibody-dependent cytotoxicity through immune activation. In the eNRGy trial evaluating zenocutuzumab, patients with *NRG1* fusion-positive solid tumors demonstrated clinical efficacy. On 4 December 2024, zenocutuzumab received FDA approval for NSCLC and pancreatic adenocarcinomas based on data from the eNRGy trial. In an updated follow-up, 158 patients across 12 unique cancer types had an ORR of 30% and a mPFS of 6.8 months [114]. The side effects were tolerable, with only one patient who discontinued due to treatment-related adverse events.

Ongoing global efforts to examine the efficacy of immunotherapy through a tumor-agnostic approach are continually being evaluated through the multicenter, open-label, multi-cohort TAPISTRY platform study [115]. Several of the aforementioned therapies including (and not limited to) entrectinib, trastuzumab, and divarasib are being studied in patients with unresectable, locally advanced, or metastatic solid tumors [115]. The TAPISTRY platform study is still underway with the primary completion estimated to be on 25 September 2032, and finalized results are expected to follow once these trials are completed [115].

The development of *KRAS*^*G12C*^ inhibitors, *FGFR* inhibitors, and *NRG1*-targeted therapies marks a significant step forward in tumor-agnostic treatments. With ongoing research, these agents have the potential to offer new, more effective treatment options for patients with diverse and difficult-to-target cancers. Additionally, other agents with novel mechanisms of action are likely to be approved in the future, further advancing precision oncology. The future of tumor-agnostic therapies is promising, with the potential for more effective and personalized cancer care.

## 7 Impact of Trial Designs in Tissue-Agnostic Targeting of Cancer Therapeutics

Tumor-agnostic therapy development is driven by innovative clinical trial designs that prioritize molecular characteristics over anatomical tumor origin. These designs enable researchers to evaluate the efficacy of therapies across diverse cancer types unified by shared genomic alterations. Unlike traditional site-specific trials, tumor-agnostic trials employ frameworks that allow for patient recruitment based on biomarkers, which accelerates drug development and enhances clinical relevance in precision oncology settings [114,116].

Master protocols have emerged as a cornerstone in the development of tumor-agnostic therapies. These include basket trials, which test a single therapy across various tumor types with the same genetic alteration (e.g., NCI-MATCH, KEYNOTE-158) [9,117], umbrella trials, which focus on one cancer type but assign patients to treatment arms based on specific molecular profiles (e.g., National Lung Matrix Trial) [118], and platform trials, which are adaptive, multi-arm studies allowing therapies to enter or exit dynamically (e.g., I-SPY 2, GBM AGILE) [119,120]. Additionally, N-of-1 trials offer highly individualized testing strategies, making them ideal for rare or ultra-rare cancers where traditional enrollment is impractical [121,122].

The NCI-MATCH trial (Molecular Analysis for Therapy Choice) is a landmark basket trial that matched patients with actionable genetic mutations to targeted therapies regardless of cancer type [9,123]. It validated the feasibility of tumor-agnostic strategies using large-scale genomic sequencing and flexible trial structures, enrolling over 1200 patients across 38 treatment arms. Importantly, it demonstrated that patients with rare cancers often have a higher prevalence of actionable mutations, supporting the inclusion of underserved populations in precision oncology [51].

Expanding on this approach, the ComboMATCH trial (launched in 2023) seeks to improve outcomes by evaluating combinatorial therapies—dual-targeted regimens or targeted drugs plus chemotherapy—to address resistance mechanisms observed in monotherapies [124]. Its trial architecture builds on NCI-MATCH but incorporates more extensive patient cohorts and adaptive mechanisms, with a particular focus on achieving durable responses. This model enhances treatment personalization and reflects a broader shift in clinical oncology toward combination-based strategies.

The TAPUR study, initiated by ASCO in 2015, complements these studies by assessing the off-label use of FDA-approved therapies in real-world clinical settings [125]. By enrolling patients with advanced cancers who have exhausted standard treatments, TAPUR evaluates molecularly targeted drugs across various tumor types. A significant outcome of TAPUR was the identification of TMB as a predictive biomarker, influencing the FDA’s approval of pembrolizumab for TMB-high cancers [8]. Collectively, these trials—NCI-MATCH, ComboMATCH, and TAPUR—demonstrate the transformative potential of trial design innovations in expanding access to and the effectiveness of tumor-agnostic therapies [124].

## 8 Conclusions

Tissue-agnostic therapies have truly transformed the way we think about cancer treatment. For decades, treatments were largely determined by where a tumor started, which often left patients with rare or difficult-to-treat cancers without many options. Now, by focusing on the genetic and molecular drivers behind cancer growth, we are not only providing more precise care but also giving hope to patients who once had very few paths forward. This shift reflects how far we have come in understanding the biology of cancer and how to fight it effectively.

That said, the journey is far from over. One of the biggest challenges is that cancers can adapt and find ways to resist even the most advanced treatments. Tumors can develop secondary mutations or activate alternative pathways, making therapies less effective over time. On top of that, the cost of these treatments and the advanced testing needed to identify eligible patients can put them out of reach for many, especially in places with limited healthcare resources. To truly make these therapies accessible to everyone who needs them, we must find ways to make testing and treatments more affordable and widely available.

The future of tissue-agnostic therapies is exciting. Researchers are working hard to discover new genetic targets, and advancements in technology like artificial intelligence are making it easier to identify which patients are likely to benefit the most. Combination therapies, which attack cancer from multiple angles, are also showing promise in overcoming resistance and improving long-term outcomes.

Tissue-agnostic therapies are a powerful reminder of what’s possible when science focuses on the unique needs of individuals. They have given us a new way to think about and treat cancer, offering personalized, targeted options that have already transformed countless lives. Once believed to be undruggable cancers have now become druggable, all thanks to the continued efforts and funding of researchers globally. Expanding the global collaborative efforts through the funding of trials will not only hasten the approval of current tissue-agnostic therapies, but acts to further preserve hope for patients without other therapeutic options. As we continue to innovate and expand access, these therapies will no doubt shape the future landscape of oncology, bringing hope and better outcomes to patients everywhere.

## Data Availability

All relevant data is contained within the manuscript: All datasets analyzed for this study are included in the manuscript.
